# Research protocol: general practice organ donation intervention—a feasibility study (GPOD)

**DOI:** 10.1186/s40814-018-0362-9

**Published:** 2018-11-12

**Authors:** Catrin Pedder Jones, Chris Papadopoulos, Gurch Randhawa, Zeeshan Asghar

**Affiliations:** 1 Institute for Health Research, University of Bedfordshire, Putteridge Bury, Hitchin Road, Luton, LU2 8LE UK; 2 NHS Blood and Transplant, 75 Cranmer Terrace, Tooting, London, SW17 0RB UK

**Keywords:** Organ donation, Intervention, Primary care, General practice, Feasibility

## Abstract

**Background:**

New interventions are required to increase the number of people donating their organs after death. In the United States of America (USA), general practice has proved to be a successful location to increase organ donor registration. However, a dearth of research exists examining this in the United Kingdom (UK). due to the unique challenges presented by the National Health Service (NHS). This protocol outlines a feasibility study to assess whether UK general practice is a feasible and acceptable location for organ donation intervention targeting NHS Organ Donor Register (NHS ODR) membership.

**Methods:**

The primary intervention element, prompted choice, requires general practice to ask patients in consultations if they wish to join the NHS ODR. Two additional intervention techniques will be used to support prompted choice: staff training and leaflets and posters. The intervention will run for 3 months (April–July 2018) followed by a period of data collection. The following methods will be used to assess feasibility, acceptability and fidelity: registration data, a training evaluation survey, focus groups with staff and online surveys for staff and patients.

**Discussion:**

By examining the feasibility, acceptability and fidelity of a prompted choice intervention in UK general practice, important knowledge can be gathered on whether it is a suitable location to conduct this. Additional learning can also be gained generally for implementing interventions in general practice. This could contribute to the knowledge base concerning the feasibility of NHS general practice to host interventions.

**Trial registration:**

International Standard Randomised Controlled Trial Number ISRTN44530504 (Jones et al, General practice organ donation intervention: a feasibility study ISRCTN44530504, 2017) Registration on 26 September 2017.

## Background

Out of the 600,000 deaths in the United Kingdom (UK) between April 2016 and March 2017, the families of 3144 people were asked if they wanted to donate their organs [1]. Over 1000 families denied this request [1]. Family consent is an important and necessary as part of the organ donation pathway and investigation into what influences family decision-making has found prior knowledge of patients’ wishes are key for these decisions [2, 3]. A formal way to acknowledge one’s wishes regarding organ donation is via the NHS Organ Donor Register (NHS ODR; [1]). Registering on the opt-in NHS ODR could help facilitate decision-making for families at a difficult time [2–4].

Currently, 36% of the population have expressed their wish to become donors in this way [1] and 44% of deceased organ donors in 2016–2017 were members of the opt-in NHS ODR [1]. In Wales, a system of presumed consent has been introduced including an opt-out register [1]. As of March 2017, 174,806 people are members of the opt-out register, equating to less than 6% of the Welsh population [1, 5]. In December 2017, the UK government opened a public consultation on whether England should introduce an opt-out system. The results of this consultation are expected in October 2018 [6]. Regardless of an opt-in or an opt-out system, it is common for families to still make the final decision on donating a loved one’s organs (often known as soft opt-out [7]). Therefore, opting in to the NHS ODR is still an important step in expressing a wish to donate organs after death, regardless of an opt-in or opt-out system of consent.

Several types of intervention targeting organ donation register sign up have been developed [8–12]. Promising results have been found in the USA involving the gate-keepers of sign-up methods, specifically the Driving Motor Vehicles Offices [13–15]. However, unlike the USA, in the UK, there is no setting in which people are routinely asked in person if they wish to join the NHS ODR [16]. The intervention in the present study aims to investigate if UK general practice could feasibly become this setting. Ninety percent of all contact with the NHS occurs in general practice [17] and it has a high footfall of patients who often return regularly for follow-up appointments [18]. Previous research has also found that GPs themselves believe primary care to be an ideal location to discuss organ donation with patients [19]. Also of note is that UK general practice is the only location within the NHS that has the ability to register patients directly on the NHS ODR [1]. An opportunity to sign-up is provided only to new patients via the new patient registration form [1]. It is proposed, therefore, that UK general practice is an ideal location to implement an organ donation intervention as it will not require large changes in NHS ODR infrastructure unlike other locations within the NHS.

To investigate this further, a systematic review investigating previous interventions targeting organ donation in a primary care setting was conducted [16]. Ten studies were found all investigating interventions in general practice. Successful interventions used ‘active’ techniques, such as volunteers approaching patients or providing registration forms to patients on check-in. ‘Passive’ techniques such as displaying posters and leaflets which patients had to approach, were less successful. Only two low-quality studies were based in the UK [20, 21]. Pradeep (2014) provided training to GP practice staff, and leaflets were displayed in the waiting room; however, this intervention resulted in no new sign-ups to the NHS ODR [20]. In contrast, a successful yet unpublished intervention was tested by Asghar et al. based on staff asking patients during their consultation if they wished to join the NHS ODR, a technique called prompted choice [21]. These studies however reported difficulties in conducting these interventions, such as lack of practice resources, time constraints and barriers to access [16]. The barriers expressed in these two studies are currently widespread in general practice in the UK [22]. GPs are under increasing pressure as the rate of recruitment to the profession does not match the growing population [22]. They experience the lowest morale amongst doctors in the NHS [23], low job satisfaction [23], experience high rates of alcohol addiction [24] and stress-related burn-out rates are higher than in other European countries [24, 25]. The effects of increasing pressure are also felt by the patients, with demand for consultations up 51% between 2007–2008 and 2014–2015 [26]. These potential barriers highlight the need for thorough investigation of general practice feasibility to host an organ donation intervention.

### Rationale

The rationale for the present study is based on the discrepancy between the number of people awaiting transplant in the UK and the current number of organ donors [27]. In an attempt to address this, interventions are required to increase the number of people registering as donors through the NHS ODR [28]. General practice interventions have previously proved to be successful in recruitment to organ donor registries in the USA [29–31] and UK [20, 21]; however, more research is required to form clear conclusions. Further, barriers to implementation and acceptability of these were expressed by GPs and other general practice staff in the UK [20, 21]. Prior to the development and evaluation of intervention efficacy in this setting, the feasibility of general practice needs to be established based on the barriers expressed. Feasibility studies investigate if the setting for the intervention is appropriate, including thorough assessment of patient views, staff views and resources required to implement it [32]. In this study, we intend to examine if general practice is a suitable place to run an intervention and if UK wide resource constraints can allow the intervention to be run successfully, consistently and acceptably for both patients and GP practice staff.

### Objective

The objective is to assess the feasibility, acceptability and fidelity of an organ donation intervention implemented in UK general practice.

## Methods

This study forms part of a PhD funded by NHS Blood and Transplant and the University of Bedfordshire. This aims to test a general practice organ donation intervention using two studies: study 1 will be a single practice feasibility study and study 2 will be a multi-practice feasibility cluster randomised controlled trial. Study 1 is outlined in this protocol, the findings of which will inform study 2.

The Standard Protocol Items: Recommendations for Interventional Trials (SPIRIT) checklist was used to structure and develop the current protocol [33]. The intervention was developed using the MRC Guidelines for Complex Interventions [34] and the Intervention Mapping method (IM: [35]). It was also developed in collaboration with the lead GP, practice manager and patient participation group (PPG) of the participating GP practice, as well as representatives of the funder (NHS Blood and Transplant) and a specialist nurse in organ donation (SNOD).

### Intervention and study design

The intervention consists of three parts: asking patients during a consultation if they wish to join the NHS ODR (prompted choice), Staff Training and Leaflets and Posters, Intervention Mapping [35] was used to develop the intervention and is a six-step process emphasising the importance of integrating theory into interventions. Following thorough theoretical review, two theories were selected which address the large intention-behaviour gap for organ donation registration [36] and the unique contextual barriers to implementation of interventions in general practice and primary care: the IIFF model of organ donation registration [36] and the conceptual framework for intervention implementation in primary care by Lau et al. (2016) [37] respectively. The former highlights four techniques which when combined in interventions lead to registration on organ donor registries: an immediate sign-up opportunity, the provision of information, focused engagement with the intervention and favourable activation (or positive affect) [36]. The latter conceptual framework provides practical recommendations for successful implementation in primary care (including general practice) across four contextual levels: external, organisational, professional and intervention contexts [37].

A single practice feasibility design will be used to assess the intervention. The intervention will be implemented in one GP practice in 2018 for a 3-month period. Following this, evaluation of the intervention will occur. This evaluation will use a mixed methods design, combining both quantitative and qualitative data collection techniques.

### Study setting

A GP practice based in Luton, Bedfordshire, agreed to take part in the intervention. The practice consists of 30 staff members, serves approximately 15,000 patients in Luton and approximately 1000 patient consultations are held each week. The intervention will be implemented in this practice, and eligible patients will be exposed to this due to their use of it.

The GP practice had previously expressed an interest in collaborating with the University on research projects. Once the University of Bedfordshire had secured funding for a feasibility study from NHS Blood and Transplant, the University approached the GP practice and presented the outline research proposal to the Lead GP, Practice Manager and the Chair of the Practice Patient Participation Group, with a view to being the GP practice for the feasibility study. The Practice agreed to this and have supported the co-design of the intervention and study.

### Intervention

#### Prompted choice

All clinical staff members (general practitioners, nurses, healthcare assistants and other clinical professionals) working in the practice, who have received training, will aim ask patients during their consultation if they wish to join the NHS ODR. Only eligible patients should be asked based on the following criteria:

1. Inclusion criteria:
  - Patient must be aged 18 years or over
  - Patient must have the capacity to consent

2. Exclusion criteria:
  (c) Patient is under 18 years of age
  (d) Patient does not have the capacity to consent

Staff will use their professional discretion to determine the capacity to consent and whether it is appropriate to ask the patient the question. They will be trained to limit potential distress by not asking those patients they believe could become distressed due to the content of their consultation. If staff are in any doubt regarding potential distress, they are advised in training not to ask the patient.

A requirement by the NHS REC is that staff must inform the patient that the question is being asked as part of a research study, a scripted question is provided to guide staff. Staff will be prompted to ask the question and to record patient responses using SystmONE, the clinical data system the practice uses. The potential responses for NHS ODR registration are based on the GMS1 form which is completed by new patients to a GP practice. The process of prompting staff and recording patient responses is described below.

Prompt Text.

*“Organ Donation Study.*

*If you will ask the patient to join the NHS ODR please press Yes.*

*If you will not ask the patient to join the NHS ODR please press No.*

*If you would like to ask the patient after the consultation, please press Pause.*

*When you are ready to ask please press ‘Save’ then ‘Resume Protocol’ or ‘Patient -> Resume’.*

*Please do not press the red x.”*

If staff member presses ‘Yes’ on the prompt, this indicates they have or will ask the patient to join the NHS ODR. They will then be instructed to complete the following questionnaire.

*“Eligibility*

1. *Does the patient have capacity to consent?*
  - *Yes*
  - *No (Staff member will be unable to complete the questionnaire if they answer no to this question)*

*Tell the following to the patient:*

*“As part of a research study, I am going to ask you some questions about organ donation.”*

2) *Patient preferences on joining NHS ODR:*
  - *Yes – Any of organs and tissues (prompt will be turned off)*
  - *Yes – Selected Organs (prompt will be turned off)*
    i. *Yes - Heart*
    ii. *Yes - Liver*
    iii. *Yes - Kidneys*
    iv. *Yes - Corneas*
    v. *Yes - Lungs*
    vi. *Yes - Pancreas*
  - *Unsure - Patient will think about it (prompt will appear again at next appointment)*
  - *Do not ask patient again (prompt will be turned off)*
  - *Patient believes they are already on the register (prompt will be turned off)*
    i. *Would the patient like to re-register?*
      1. *Yes - All organs and Tissues*
      2. *Yes - Selected organs and Tissues*
        i. *Yes - Heart*
        ii. *Yes - Liver*
        iii. *Yes - Kidneys*
        iv. *Yes - Corneas*
        v. *Yes - Lungs*
        vi. *Yes - Pancreas*
      3. *No - Patient would not like to re-register*
  f. *Patient was not asked*
    i. *Not appropriate for consultation*
    ii. *Lack of time*
    iii. *Clinician personal beliefs (only select if personal view of clinician NOT situational factor)*
    iv. *Other reason, please specify*

If staff member presses ‘No’ on the prompt, this indicates they have not or will not ask the patient to join the NHS ODR. They will then be instructed to complete the following questionnaire which records reason why the patient was not asked.

g. *Patient was not asked. Reason why patient was not asked:*
  i. *Not appropriate for consultation*
  ii. *Lack of time*
  iii. *Clinician personal beliefs (only select if personal view of clinician prevents patient being asked)*
  iv. *Other reason, please specify*

It was highlighted that staff may select the ‘Yes’ questionnaire at the beginning of a consultation but be unable to carry out prompted choice as the consultation progresses. To allow staff to record that the patient was not asked under these circumstances question f. was included in both the ‘Yes’ and ‘No’ questionnaire.

To ensure no untrained staff ask patients about organ donation, the prompt will only appear for staff who have received the training and only for patients aged 18 or over. If prompted choice has previously been completed for the patient and the patient responded “Yes”, “Do not ask patient again” or “Patient believes they are already on the register”, the prompt will switch off and the patient will not be asked again at subsequent appointments.

#### Training

Training will be conducted prior to the prompted choice commencement and will be provided by the research team, NHS Blood and Transplant (NHSBT) and the specialist organ donation nurse training division. Clinical staff, experienced in having organ donation conversations with grieving families, will train practice staff on how to discuss the organ donor register with patients. The specific training content will be provided by NHSBT. Training in the previously described adaptations made to the computer system (SystmONE) will be provided. This will also outline the computer-based quantitative data collection methods.

#### Leaflets and posters

Existing NHSBT leaflets and posters will be displayed in an area to be determined by the practice. These will help prompt patients to think about organ donation prior to the question being asked in their consultation. Additionally, staff can direct patients to the leaflets which contain organ donation information for those undecided. The leaflets, posters and languages to be displayed are those published by NHSBT at the time of intervention. Leaflets may contain registration forms which patients could complete in the practice. If patients request to hand these in at reception, staff can place these into a lockable opaque box which only the research team can access.

### Participant timeline, sample size and recruitment

Patients will participate in the intervention by their use of the practice during the 3-month prompted choice period (5 April 2018–9 July 2018). Participants will enter the practice and be exposed to the study leaflets and posters. They will then be asked by the trained staff member taking the consultation if they wish to join the NHS ODR (subject to inclusion and exclusion criteria and staff member discretion of appropriateness). The patient could be directed to the leaflets by the staff member if they request more information and may pick these up when leaving the practice. A sample size calculation was not conducted for this study due to its nature as a first stage feasibility study [34]. A second study not described in the protocol aims to collect the data required to inform a sample size calculation for a future full randomised controlled trial. However, it is anticipated during a 3-month trial period that approximately 10,000 patient consultations will occur. This is dependent on staffing in the practice during this time period. Table 1 presents the SPIRIT figure to outline this timeline.

**Table 1:** SPIRIT figure

	Study period					
	Site enrolment	Intervention and data collection period				
Timepoint	*-t*_*1*_	April 2018	May 2018	June 2018	July 2018	August 2018
Enrolment	X					
SystmONE Setup	X					
INTERVENTIONS:						
Staff training	X					
Leaflets and posters displayed		X	X	X	X	
Patients asked question (if eligible)		X	X	X	X	
ASSESSMENTS:						
Training evaluation	X					
Registration data		X	X	X	X	
Focus groups					X	
Staff online survey					X	X

### Monitoring

During the 3-month prompted choice period, the intervention will be monitored weekly by the research team. This will include an examination of raw data from SystmONE to ensure no staff member without training has conducted prompted choice, restocking leaflets and ensuring all informative posters are still in place. If it is found that an untrained staff member has asked a patient the question, due to computer error, the investigator will inform them verbally that they should not be doing this and aim to amend the prompt on SystmONE.

### Data collection, management and analysis

Data will be collected in four ways: registration data via the practice computer system, a training evaluation survey, focus groups with staff and patients and an online survey with participating staff.

### Registration data

The data to be collected through SystmONE can be viewed in Table 2. Any leaflet registrations collected in the practice will be included in registration data analysis. However, it is possible that patients may register on the NHS ODR and post these forms themselves. Therefore, registration by leaflet data, collected at reception, will be treated with caution during analyses and interpretation.

**Table 2:** Registration data to be collected by SystmONE

Variable	Response format
Registration type	SystmONE/Paper
Date of consultation	Dd/mm/yy
Patient age	Years
Patient ethnicity	ONS categories
Patient gender	Male or female
Patient asked	Yes/no
Reason patient not asked	Not appropriate/time/clinician personal beliefs/other (free text)
Patient joined NHS ODR	Yes/no/patient believes already on register
Organs selected	All/kidneys/heart/liver/corneas/lungs/pancreas
If no, which response	Unsure/do not ask again
Patient believes already on register. Re-register	Yes/no
Organs selected	All/kidneys/heart/liver/corneas/lungs/pancreas
Staff group	Doctor/Nurse/Healthcare Assistant

### Training evaluation survey

To examine staff reaction to the training, a qualitative and quantitative paper survey will be distributed directly after it has been delivered. Participants will be provided with an information sheet, consent form (referring to the training evaluation survey only) and training evaluation form. Confidentiality, anonymity, the voluntary nature of the survey and the right to withdraw will be highlighted to participants prior to them completing the consent forms. The evaluation form will quantitatively assess overall views of the session, how prepared the staff feel to conduct the prompted choice element and answer questions about organ donation, the acceptability of the training content, presentation style and use of resources. Qualitative open questions will be asked concerning strengths, weaknesses and potential improvements to the training session.

### Staff focus groups

#### Participants and recruitment

Following completion of the prompted choice period, participating staff will be invited to take part in qualitative focus groups. Each focus group will contain 6–10 participants and will be split according to staff group (clinical GP, clinical non-GP and administrative). Staff will be invited via email and an information sheet will be attached. The focus groups will take place for 1 h, during the 2-h (12 pm–2 pm) staff lunch break to minimise the impact on the running of the practice, with lunch provided for staff by the research team. To ensure staff are able to attend the focus groups, these will be organised to maximise staff availability.

#### Materials and procedure

Focus groups will take place in the practice, and a digital audio recorder will be used to record the sessions. Prior to commencement, participant information sheets will be distributed again alongside consent forms. These outline that all responses are confidential, anonymous and they have the right to withdraw at any time. Once consent has been sought, the focus groups will commence and topic guides will guide the focus group discussion. Questions will be asked by a facilitator concerning staff and patient experiences of each aspect of the intervention (training, prompted choice and leaflets and posters), staff perceptions of impact on the day to day running of the practice and recommendations. These topic guides have been devised based on the IIFF model of organ donation registration [36] and the conceptual framework for implementing interventions in primary care by Lau et al. (2016) [37]. Following the focus groups, participants will receive a verbal debrief and be referred to the contact details for the research team and telephone numbers for bereavement support.

### Patient online survey

To capture patient experiences of the intervention, an online survey will be conducted with patients.

#### Participants and recruitment

A qualitative online survey will be distributed to patients who are over 18 years of age, are patients who attended the practice for consultation within the 3-month intervention period and for whom a ‘Yes Questionnaire’ is completed. The practice will send an advertisement text message containing a link to the questionnaire to all patients registered to receive text messages from the practice.

#### Materials and procedure

The online survey will be hosted on Qualtrics (2018) [38] and consist of nine open questions and three closed questions. Prior to answering these questions, patients will be presented with an information sheet, consent form and demographic questionnaire. Consent will be gained via ticking boxes on Qualtrics (2018) [38], and the survey should take approximately 10 min to be completed, determined through piloting by the research team.

### Staff online survey

As well as focus groups, an online survey will be distributed to staff. Focus groups capture collective views and group interactions. However, organ donation is a personal topic and some staff may prefer to express opinions privately than in a group. An online survey will help elicit as many views of the intervention in terms of feasibility as possible.

#### Participants and recruitment

Following completion of the focus groups, an online survey will be distributed to all trained staff members. No incentives will be used to recruit staff; however, a reminder email will be sent approximately 2 weeks following the first email. A final reminder email, with the same content, will be sent at 4 weeks following the first email. Staff will be informed that this survey aims to capture any experiences or views that they do not feel comfortable sharing in the focus groups or anything they forgot to mention during the focus group.

#### Materials and procedure

The online survey will be qualitative and consist of 13 open questions. Prior to answering these questions, staff will be presented with an information sheet and consent form. Consent will be gained via ticking boxes on Qualtrics and the survey should take approximately 10 min.

### Data management

Data will be accessed through SystmONE at the practice by the investigator. Only data relevant to the study (i.e. the data specified in Table 2) will be accessed. Data will be anonymised by the investigator and exported from SystmONE for analysis on an encrypted memory stick. Paper consent forms and training evaluation forms will be stored in a locked filing cabinet at the University of Bedfordshire with access only available to the investigator. These will be stored for 5 years. Online staff survey data will be securely hosted on Qualtrics, who adhere to the data protection act, the general data protection regulation and online data protection procedures. At no point will participants be asked their name, and the only identifiable data collected by Qualtrics will be participants IP address. This will be deleted in all data sets extracted for analysis.

### Data analysis

#### Quantitative analyses

Quantitative analyses will be conducted using SPSS version 23 [39] to explore the variables listed in Table 1. Including the demographics of those asked and not asked, actual registration rates, and whether any differences in these are related to staff group. These analyses will be descriptive in nature and include measures of central tendency. Assessment of baseline recruitment rates to the ODR by the practice (for 1 year prior to the intervention through new patient registration forms) will be compared to the total registration rates in the intervention period. This data will be supplied by the practice administrator.

#### Qualitative analyses

All recorded focus group data will be transcribed verbatim by the investigator and inputted into NVivo 11. Staff online and patient survey responses and training evaluation open questions will be inputted also. All qualitative data captured will be analysed via framework analysis [40]. Framework analysis allows for themes to be compared across the medium they were captured and by their participant group. In this instance, by method (focus group, online survey or training evaluation) and by participants (patients or staff) [40]. Themes will be categorised based on the underpinning theoretical framework for this study, Lau et al.’s (2016) conceptual framework for implementation of primary care interventions and the IIFF Model of Organ Donation Registration [36, 37].

### Ethics and dissemination

#### Consent

As previously specified, formal informed consent will be sought from staff prior to their involvement in training evaluation, focus groups and the online survey, once the intervention has ceased. Informed consent will also be sought from patients prior to their involvement in the online survey. The Information Sheets for each focus group, online survey and consent forms address the key consent principles highlighted by the HRA will ensure participants understand the purpose and nature of the research, what the research involves, its benefits (or lack of benefits), risks and burdens, the alternatives to taking part, how long data will be stored for, that participation is voluntary and that they are able to make a decision on consent at the time of the focus group.

Overt informed consent, to collect and use prompted-choice patient data for the purposes of this research, will not be sought for each patient with whom prompted choice occurs. This is due to the practical difficulties of obtaining formal consent in each consultation, in a working GP practice over a 3-month period. This issue was discussed in-depth with the practice, particularly the patient participation group who approved the use of patient data in this way. The data collected will be gender, age, ethnicity, registration status and organs chosen for donation. Additional data collected concerning the consultation will not be related to the patient and only concern feasibility measures.

#### Anonymity and confidentiality

Focus group discussions will be recorded via digital audio recorder and the transcription of these will be anonymised. Recordings will be stored on encrypted USB in a locked drawer for 5 years before being destroyed. Participants will be informed that all responses will be confidential and anonymised and that responses may be traceable back to the practice in publication. Any quotes published will not refer to participants by name. This will also be stipulated for staff completing the online survey.

#### Distress

For staff and patient well-being, the contact details for bereavement support services will be displayed throughout the practice. Staff will be advised to direct patients to these contact details and staff will be informed that they can refrain from having conversations if it will cause them distress. If staff do not have the conversation for these reasons, this will be recorded to ensure accurate assessment of feasibility can be conducted and take into account the element of distress.

#### Coercion

In the context of joining the organ donation register, it is vital that any patient decision made is elective. Therefore, staff will be trained that, as opposed to normal clinical practice, they are not advising or recommending a patient join the register, merely offering them the opportunity if they wish to. This will be a significant part of staff training and staff will be given scripted examples to help guide them in the appropriate language to use to avoid coercion. Additionally, if they believe the patient may feel under pressure to join, they will be advised to stop the conversation and ask the patient to think about it. As above, if this occurs, it will be recorded on to SystmONE by the GP.

#### Impact on normal running of the practice

The intervention will be mindful of the normal running of the practice. Ensuring the impact on the working practice is minimal has been achieved by collaborating with the practice and patient group throughout the design. Staff will be informed that the priority is patient care, and the intervention should always come second.

#### Dissemination policy

Research findings will be presented in various publications throughout this study. Namely, conference presentations, academic journals and media releases. The intention being is to inform future researchers of the success of implementing an organ donation intervention in a general practice setting.

## Discussion

Membership of the NHS ODR is the primary way to express organ donation wishes in the UK [1]. Interventions to target this are vital in reducing the discrepancy between the number of people donating their organs after death and those on the transplant list [1]. Currently, there is no location in the UK where people are routinely asked if they would like to join the NHS Organ Donor register. This study proposes general practice could be this location. However, the NHS is currently experiencing significant challenges [16]; therefore, an assessment of feasibility is essential before examining if this intervention would increase NHS ODR membership.

A limitation of this research is the lack of incentives for both the GP practice as a whole and for the individual participation of staff and patients in prompted choice. Introduced in 2004, the Quality Outcomes Framework (QOF) provides practices with financial reimbursement for each patient asked about a variety of health problems; for example smoking cessation [41]. Therefore, the lack of incentive in the current intervention may decrease the likelihood of staff completing the intervention form, including the elements concerning feasibility (e.g. reasons for not asking patients) and reduce the data captured using this method. Focus groups and online surveys could help examine the impact of this on the intervention; however, recruitment to these may also be impacted by the limited incentives (lunch for staff).

A key ethical concern when developing this research is the issue of distress to patients and staff during organ donation conversations. Predicting who might become distressed by the prompted choice conversation is challenging. To help ensure as minimal distress to patients occurs as possible, staff will be trained in how to best have these conversations by the experienced specialist organ donation nurse training division, a part of NHS Blood and Transplant. These staff have these conversations daily with grieving families and will help guide staff in this. Additionally, staff will be informed that they must use their professional judgement as to whether a conversation is appropriate for the patient they are about to see. If staff are unsure, they will be instructed to refrain from asking the question.

In contrast, a strength of the proposed study is the current lack of investigation of UK general practice to target organ donation registration [16]. The contribution to knowledge therefore will be to examine if general practice is appropriate for this. A further strength is the involvement of the GP practice throughout the development of the intervention and data collection. Additionally, the study is underpinned by strong theory and methodology. Intervention Mapping is a well-respected method of intervention development and allows the integration of behaviour change theory into interventions an important and often neglected step by intervention developers [35]. Throughout the process, Lau et al.’s conceptual framework was used to design the implementation of the intervention. This framework specifies the importance of context within implementation and provides overt recommendations specifically tailored to the primary care setting [37]. The IIFF model, also used to underpin the intervention, was developed iteratively from interventions designed to increase organ donor registrations. It provides a practical specification for intervention compared to other socio-cognitive models of behaviour change which has more abstract components which are difficult to translate into actual interventions ([42]; e.g. self-efficacy, social norms, attitudes and intention from the Theory of Planned Behaviour TPB).

Finally, the study described in the present protocol is the first of two studies to examine the feasibility of this organ donation intervention in UK general practice. Initial exploration in a single GP practice allows for refinement of the intervention using Intervention Mapping prior to further feasibility testing. The findings from this study will inform a multi-centre feasibility cluster randomised controlled trial, which will aim to explore the recruitment of GP practices and collect data to perform a sample size calculation for full randomised controlled trial.

## Abbreviations

CAG: Confidentiality Advisory Group
HRA: Health Research Authority
IM: Intervention Mapping
NHS ODR: NHS Organ Donor Register
NHS REC: NHS Research Ethics Committee
NHSBT: NHS Blood and Transplant
PPG: Patient Participation Group
QOF: Quality Outcomes Framework
SNOD: Specialist Nurse in Organ Donation
SPIRIT: Standard Protocol Items: Recommendations for Interventional Trials
TPB: Theory of Planned Behaviour

## References

[1] NHS Blood and Transplant (2017). Organ Donation and Transplantation. Activity report 2016/2017.

[2] de Groot J, van Hoek M, Hoedemaekers C, Hoitsma A, Smeets W, Vernooij-Dassen M (2015). Decision making on organ donation: the dilemmas of relatives of potential brain dead donors. BMC Med Ethics.

[3] Ralph A, Chapman JR, Gillis J, Craig JC, Butow P, Howard K (2014). Family perspectives on deceased organ donation: thematic synthesis of qualitative studies. Am J Transplant.

[4] Hulme W, Allen J, Manara AR, Murphy PG, Gardiner D, Poppitt E (2016). Factors influencing the family consent rate for organ donation in the UK. Anaesthesia.

[5] Welsh Goverment. Mid year estimates of the population. 2017. http://www.gov.wales/statistics-and-research/mid-year-estimates-population/?skip=1&lang=en. Accessed 1 Nov 2018.

[6] Department of Health and Social Care (2017). Introducing ‘opt-out’ consent for organ and tissue donation in England.

[7] Rithalia A, McDaid C, Suekarran S, Norman G, Myers L, Sowden A (2009). A systematic review of presumed consent systems for deceased organ donation. Health Technol Assess.

[8] Li AH, Rosenblum AM, Nevis IF, Garg AX (2013). Adolescent classroom education on knowledge and attitudes about deceased organ donation: a systematic review. Pediatr Transplant.

[9] D’Alessandro AM, Peltier JW, Dahl AJ (2012). A large-scale qualitative study of the potential use of social media by university students to increase awareness and support for organ donation. Prog Transplant.

[10] Ramadurg UY, Gupta A (2014). Impact of an educational intervention on increasing the knowledge and changing the attitude and beliefs towards organ donation among medical students. J Clin diagnostic Res JCDR.

[11] Kurz JM (2014). Impact of organ donation education on US undergraduate nursing students. Prog Transplant.

[12] Douville F, Godin G, Vézina-Im L-A (2014). Organ and tissue donation in clinical settings: a systematic review of the impact of interventions aimed at health professionals. Transplant Res.

[13] Rodrigue JR, Krouse J, Carroll C, Giery KM, Fraga Y, Edwards E (2012). A Department of Motor Vehicles intervention yields moderate increases in donor designation rates. Prog Transplant.

[14] Zaramo CEB, Morton T, Yoo JW, Bowen GR, Modlin CS (2008). Culturally competent methods to promote organ donation rates among African-Americans using venues of the Bureau of Motor Vehicles. Transplantation proceedings.

[15] Harrison TR, Morgan SE, Di CMJ (2008). Effects of information, education, and communication training about organ donation for gatekeepers: clerks at the Department of Motor Vehicles and organ donor registries. Prog Transplant.

[16] Jones Catrin Pedder, Papadopoulos Chris, Randhawa Gurch (2017). Primary care interventions to encourage organ donation registration: A systematic review. Transplantation Reviews.

[17] Hippisley-Cox J, Vinogradova Y (2009). Final report to the NHS Information Centre and Department of Health Trends in consultation rates in general practice 1995/1996 to 2008/2009: analysis of the QResearch® database.

[18] Lock C, Wilson G, Kaner E, Cassidy P, Christie M, Heather N (2009). A survey of general practitioners’ knowledge, attitudes and practices regarding the prevention and management of alcohol-related problems: an update of a World Health Organisation survey ten years on.

[19] Rea J, Meakin R. A new means to promote organ donation: Doctors' views on discussing organ donation in the primary care setting - a qualitative study.

[20] Pradeep A (2014). Increasing organ donation from the North West South Asian community through targeted education.

[21] Asghar Z, NHS Blood and Transplant. GP Surgeries Trial - ‘Prompted Choice’: Increasing registrations to the NHS Organ Donor Register.

[22] Baird B, Charles A, Honeyman M, Maguire D, Das P (2016). Understanding pressures in general practice. King’s Fund.

[23] Croxson CHD, Ashdown HF, Hobbs FDR (2017). GPs’ perceptions of workload in England: a qualitative interview study. Br J Gen Pr.

[24] Brooks SK, Gerada C, Chalder T (2011). Review of literature on the mental health of doctors: are specialist services needed?. J Ment Health.

[25] May CR, Johnson M, Finch T (2016). Implementation, context and complexity. Implement Sci.

[26] Hobbs FDR, Bankhead C, Mukhtar T, Stevens S, Perera-Salazar R, Holt T (2016). Clinical workload in UK primary care: a retrospective analysis of 100 million consultations in England, 2007–14. Lancet.

[27] NHS Blood and Transplant (2017). Organ donation and transplantation – activity figures for the UK as at 7 April 2017.

[28] NHS Blood and Transplant (2008). Organs for Transplants: A report from the organ donation taskforce.

[29] Thornton JD, Sullivan C, Albert JM, Cedeno M, Patrick B, Pencak J (2016). Effects of a video on organ donation consent among primary care patients: a randomized controlled trial. J Gen Intern Med.

[30] Salim A, Berry C, Ley EJ, Schulman D, Anderson J, Navarro S (2014). Improving organ donor registration using kiosks in primary care clinics. Health Educ J.

[31] Bidigare SA, Ellis AR (2000). Family physicians’ role in recruitment of organ donors. Arch Fam Med..

[32] Tickle-Degnen L, C J, LR V, TH D, A M, de GCJ T (2013). Nuts and bolts of conducting feasibility studies. Am J Occup Ther.

[33] Chan A-W, Tetzlaff J, Altman D, Laupacis A, Gøtzsche P, Research K-JK (2013). Reporting methods annals of internal medicine SPIRIT 2013 statement: defining standard protocol items for clinical trials. Ann Intern Med.

[34] Craig P, Dieppe P, Macintyre S, Michie S, Nazareth I, Petticrew M (2013). Developing and evaluating complex interventions: the new Medical Research Council guidance. Int J Nurs Stud.

[35] Eldredge LKB, Markham CM, Kok G, Ruiter RAC, Parcel GS (2016). Planning health promotion programs: an intervention mapping approach.

[36] Siegel J, Alvaro E, Hohman Z. A dawning recognition of factors for increasing donor registration: the IIFF model. Underst Organ Donation Appl Behav Sci Perspect. 2010:313–30.

[37] Lau R, Stevenson F, Ong BN, Dziedzic K, Treweek S, Eldridge S (2016). Achieving change in primary care—causes of the evidence to practice gap: systematic reviews of reviews. Implement Sci.

[38] Qualtrics. Qualtrics. 2018.

[39] IBM Corp (2015). IBM SPSS Statistics for Mac, Version 23.0.

[40] Gale NK, Heath G, Cameron E, Rashid S, Redwood S (2013). Using the framework method for the analysis of qualitative data in multi-disciplinary health research. BMC Med Res Methodol.

[41] Downing A, Rudge G, Cheng Y, Tu YK, Keen J, Gilthorpe MS (2007). Do the UK government’s new Quality and Outcomes Framework (QOF) scores adequately measure primary care performance? A cross-sectional survey of routine healthcare data. BMC Health Serv Res.

[42] Sniehotta FF, Presseau J, Araújo-Soares V (2014). Time to retire the theory of planned behaviour. Health Psychol Rev.

[43] Jones CP, Papadopoulos C, Randhawa G. General practice organ donation intervention: a feasibility study ISRCTN44530504. ISRCTN Regist 2017. 10.1186/ISRCTN44530504. 10.1186/s40814-018-0362-9 PMC6231272 30459960

